# Wear-adaptive optimization of in-process conditioning parameters during face plunge grinding of PcBN

**DOI:** 10.1038/s41598-022-05066-5

**Published:** 2022-01-19

**Authors:** Berend Denkena, Alexander Krödel-Worbes, Dominik Müller-Cramm

**Affiliations:** Institute of Production Engineering and Machine Tools, An der Universität 2, 30823 Garbsen, Germany

**Keywords:** Mechanical engineering, Aerospace engineering, Design, synthesis and processing

## Abstract

Polycrystalline cubic boron nitride is a very hard material. Machining of this material is performed by grinding with diamond tools. Due to its high hardness, grinding tools are subjected to severe microscopic and macroscopic tool wear. This wear leads to short tool life and results in high effort in conditioning the abrasive layer. Contrary to the usual conditioning of diamond grinding wheels with diamond dressing tools, this study investigates a conditioning process based entirely on the use of white corundum cup rolls. These conditioning tools allow the in-process face plunge conditioning of vitrified bond diamond grinding tools. The circumferential speed of the conditioning tool and the average grain diameter of the corundum are identified as the main factors influencing the topography of the generated grinding layer. To describe the performance of the conditioning process, a specific conditioning removal rate $Q_{sd}^{\prime }$ is derived. This parameter represents a cumulated variable that allows a comparison of different conditioning strategies. It is shown that an increase in $Q_{sd}^{\prime }$ significantly counteracts microscopic wear on the abrasive layer. Therefore, optimized process parameters enable the process of in-process conditioning to significantly reduce wear on the grinding tool without increasing the process time or the non-productive time.

## Introduction

The manufacturing process grinding enables the machining of brittle hard materials. In this case, the stochastic distribution and shape of the cutting edges in the grinding tool are used by accepting the wear of the grinding tool as a loss variable. Cutting edges are formed by abrasive grains that are held in the abrasive layer by a bonding material. Abrasive layers with multiple levels of abrasive grains aim to regenerate the worn abrasive surface. Grains worn by the grinding process are removed from the layer and the underlying grain takes over material removal. Therefore, the second hardest material after diamond, the polycrystalline boron nitride (PcBN), is also machined mainly by grinding^1^. PcBN consists of cubic crystalline boron nitride (cBN) as the hard material phase and a binder. The high thermomechanical strength of PcBN is mainly determined by the material properties of the hard material phase. With increasing cBN content, the hardness of PcBN increases^2–4^. cBN has a cubic structure and reaches a hardness of up to 5,000 HV for a single crystal^5^. Compared to diamond, cBN is thermally stable at temperatures *T* < 1200 °C. Its hardness at these temperatures is above 900 HV^6^. Therefore, PcBN is suitable as a cutting material for geometrically determined hard machining. Thus, PcBN can be used as a cutting material at higher temperatures at the cutting edge than coated carbide tools with typical coating systems AlTiN, TiAlN, or TiCN^7^. Common binder materials are AlN and TiN or mixed materials such as TiCN^2^.

The final shaping of the cutting material PcBN could only be achieved by cutting processes with geometrically undefined cutting edges, such as grinding. The extreme hardness of PcBN (2600–5500 HV^2–4^) presented a particular challenge for grinding operations. As a result of its high hardness, PcBN can only be economically ground with diamonds as abrasive grains due to the even higher hardness of 8150 HV^5^. The grinding of flat surfaces of PcBN is often performed by face plunge grinding. The face plunge grinding process enables a high flatness of the machined surfaces by using flat-faced cup grinding wheels. In prior studies abrasive layers with diamonds of small grain size *d*_*g*_ < 20 µm with a highly porous vitrified bond resulted in high manufacturing quality and low tool wear. Using the small grain size of the abrasive, low roughness values *Rz* < 1 µm, *Ra* < 0.4 µm have been achieved on ground surfaces^1,8^. The low surface roughness was attributed to the low single grain chip thickness for a small grain size *d*_*g*_. However, the influence of the process parameters cutting speed *v*_*c*_ and feed rate *v*_*fa*_ on the surface roughness was not significant^8^. Although according to relevant models the single grain chip thickness should also decrease with an increase of the cutting speed^1,9^. The vitrified bond is mechanically resistant and, due to its brittle fracture behavior, can be dressed by mechanical processes. The dressing ensures the flatness of the abrasive layer. For dressing diamond grinding tools with rotating profiling tools, diamond rollers or rollers made of vitrified bonded SiC are mainly used as rotating dressing tools. When dressing with diamond profiling tools, the profile of the abrasive layer can be adjusted in a wide range of applications^10^. In particular, CVD diamond form rolls enable high profile accuracy when dressing vitrified bonded conventional and superabrasives. However, this method is only suitable to a limited degree for producing flat profiles with high flatness, as the profile of the dressing tool is imprinted on the surface of the abrasive layer. If a low relative speed is applied between the dresser and the grinding tool, fractures may also occur in the vitrified bond. These fractures reduce the retention force of the bond against the abrasive grain and thus the tool life^11^. The dressing is usually performed on specially designed dressing machines with high rigidity. Dressing in the grinding machine tool is also possible, but extends the non-productive time^10^.

Huang defined the dressing intensity as the average force applied to each abrasive grain during dressing. In the study, the influence of the dressing process with both SiC and mild steel dressing rollers on a vitrified bonded diamond wheel was investigated. The dressing was performed in the non-productive time. Huang showed that the grinding forces decrease as the dressing intensity increases, and he concluded that a high dressing intensity generated larger and sharper cutting edges. When grinding with those sharp cutting edges, there were lower grinding forces. This was attributed to fewer cutting edges per individual grain. In contrast, a low dressing intensity resulted in multiple micro cutting edges and therefore increased grinding forces^12^.

For high wear rates, continuous dressing can be used to increase manufacturing quality. This dressing process is performed within the primary processing time. Continuous dressing demands constant contact between the dressing tool and the abrasive layer. Investigations in in-process dressing by Wegener et al. showed constant grinding forces and grinding wheel profile during the grinding process^13^. Deng et al. stated, that electronlytic in-process dressing (ELID) is applied to metal bonded superabrasive grinding tools, because this process needs a conductive grinding layer. For non-conductive bonds the application of ultrasonic assisted mechanical dressing was described as efficient cleaning of the surface^14^.

The tool wear and energy consumption in face plunge grinding of PcBN was high regarding the specific material removal $V_{w}^{\prime }$. Distinct microwear on the diamond grains was observed as dulling of the grains. As a result, the abrasive grains had reduced cutting ability, leading to increased local forces and subsequent grain breakout. This breakout led to high profile wear of the abrasive layer on the macrolevel. In addition, the dulled cutting edges of the diamond grains led to an increased proportion of friction and ploughing in the process. Consequently, a major share of the cutting energy was converted into heat, and the required cutting power for the material removal increased significantly with each tool engagement. Previous studies have shown that the specific cutting energy increased by *e*_*c*_ = 500 J/mm^3^ per $V_{w}^{\prime }$ = 1 mm^3^ of ground PcBN. In conclusion, the energy consumption of the process and the tool wear increased significantly with increasing $V_{w}^{\prime }$^15,16^. Therefore, the grinding tool has to be cleaned and sharpened to maintain its process capability.

The sharpening process resets the bond of the abrasive layer and increases the grain protrusion of the diamonds. In addition, sharpening leads to cleaning of the abrasive layer from potential clogging and welds. It was shown that abrasive grains could also release from the bond if the grain protrusion exceeded a critical value. Sharpening can be performed mechanically, electrochemically, or by spark erosion^17^. However, electrochemical and spark erosion processes require an electrically conductive grinding layer^18^. Therefore, these sharpening processes could not be applied the sharpening of nonconductive vitrified bonded grinding tools. In contrast, mechanical conditioning processes can also be used for nonconductive abrasive layers with vitrified or resin bond. The bond removal is either energy-related or path-related. In energy-related processes, an abrasive is blasted onto the surface of the grinding tool, causing the removal of the bonding material. In path-related processes, sharpening is performed by vitrified white corundum. Sharpening with white corundum is carried out with stationary sharpening tools, block sharpening. The abrasive layer is moved into contact with the sharpening block by a translatory movement. As the hardness of the diamond is higher than the hardness of the conventional abrasive, the diamond is not or little damaged. However, the bond and impurities on the surface of the grinding tool are removed^10,18^.

A novel conditioning process for plunge face grinding is plunge face dressing with cup conditioning tools made of vitrified bonded white corundum. These conditioning tools can be used to condition vitrified bonded diamond grinding tools in non-productive time and in-process. Although this method is used in grinding machine tools from leading machine manufacturers, there have been no scientific studies on in-process conditioning. Previous studies of conditioning mainly focused on the effects of conditioning parameters on the grinding result of conditioning in non-productive time^19^. The influence of the conditioning process on the grinding wheel topography, tool wear, and the subsequent grinding process has not yet been adequately investigated. Therefore, model-based process parameterization is not yet available. The present investigations aim to provide a model-based conditioning strategy that allows wear-adapted in-process conditioning of vitrified diamond grinding tools during PcBN machining.

## Experimental setup and methods

The test series aims to investigate the influence of the conditioning of vitrified bonded diamond grinding wheels on the grinding wheel wear, respectively, the specific grinding power. Therefore, the influence of the process parameters and the conditioning tool specification on the grinding tool topography are investigated.

**Figure 1:**
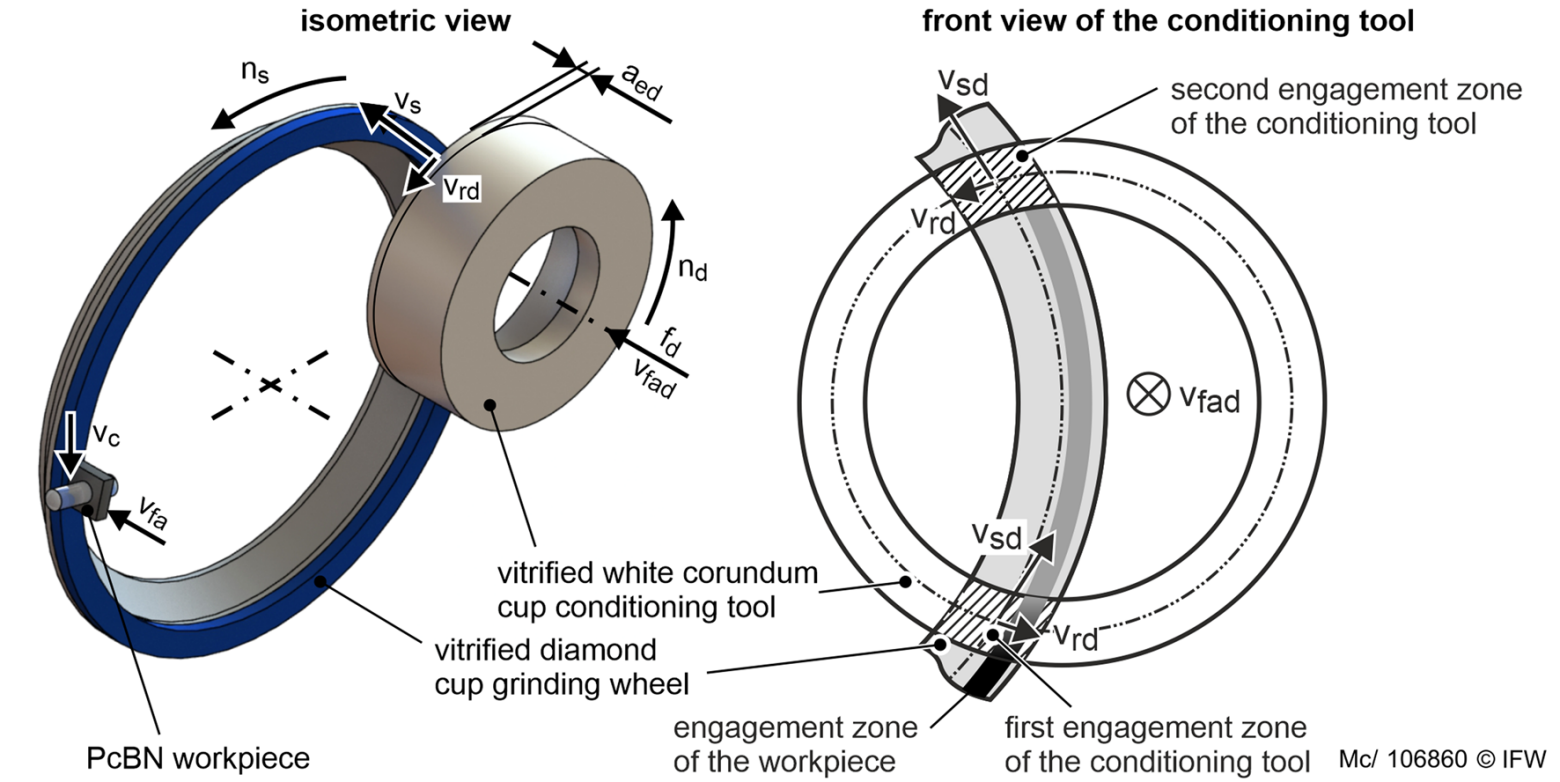
Kinematics of in-process conditioning during face plunge grinding. The cup conditioning tool is mounted parallel to the surface of the cup grinding wheel.

The investigations are carried out on a Wendt WAC 715 Centro cutting insert grinding machine. This machine is equipped with a RotoDress conditioning system. In contrast to the usual dressing systems, which operate with diamond or SiC discs, this conditioning system uses cup dressing rolls made of vitrified bonded white corundum. The conditioning system allows the conditioning in the non-productive time and in-process conditioning during grinding (Fig. 1, left). The rotational movement is performed by a hydraulic motor with a rigid structure. The rotational speed can be varied in the range *n*_*d*_ = 220–600 rpm, which corresponds to a circumferential speed of the conditioning tool of *v*_*rd*_ = 1.5–3.5 m/s.

When dressing in the non-productive time, there is no contact between the workpiece and the grinding tool. The grinding tool rotates at the rotational speed *n*_s_and the conditioning tool at the rotational speed *n*_*d*_. The conditioning tool is fed by an axial feed axis at a feed frequency *f*_*d*_ by the depth of cut per conditioning stroke *a*_*ed*_. On each conditioning stroke, the conditioning tool is rapidly moved against the grinding layer by depth of cut between *a*_*ed*_ = 0.5–1.5 µm. Hence, the average axial infeed rate is the product of *a*_*ed*_ and *f*_*d*_ the strokes are performed. During in-process conditioning, the grinding tool engages the workpiece and the conditioning tool simultaneously. The workpiece is moved at the axial feed rate *v*_fa_ against the abrasive layer normal to the flat abrasive layer surface. Contrary to common dressing processes with cup dressing tools, the aim is to achieve an areal contact between the dresser and the grinding layer (Fig. 1, right). The infeed movement of the cup conditioning tool is directed normally to the surface of the grinding layer. The planar alignment of the faces of the grinding and conditioning tool ensures the areal contact and thus a self-compensating axial run-out of both tools. Therefore, there are two engagement zones between the grinding layer surface and the cup conditioning tool. As a result of the two engagement zones, the direction of *v*_*rd*_ is crossed regarding the direction of *v*_*s*_. Thus, a corundum grain of the conditioning tool engages in the first engagement zone at the left edge of the abrasive layer and in the second engagement zone at the right edge of the abrasive layer.

**Figure 2:**
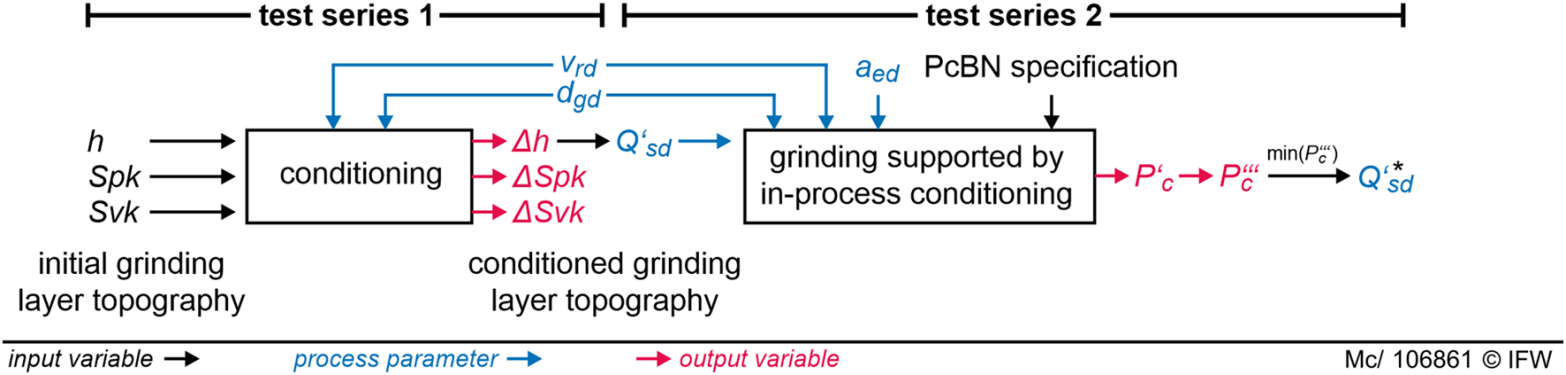
Dependencies between parameters and variables in the test series.

To investigate the influence of circumferential speed, corundum grain size, and cut depth in conditioning, two series of tests are performed (Fig. 2). Input variables in test series 1 are topography parameters of the grinding surface. In all test series, the grinding layer is conditioned with the same parameters to create an unworn state. Thus, constant starting conditions are provided for the investigation. For this purpose, 100 strokes with an infeed *a*_*ed*_ = 1 µm each are performed each using a vitrified white corundum cup dresser with a grain size of *d*_*gd*_ = 48 µm.

**Figure 3:**
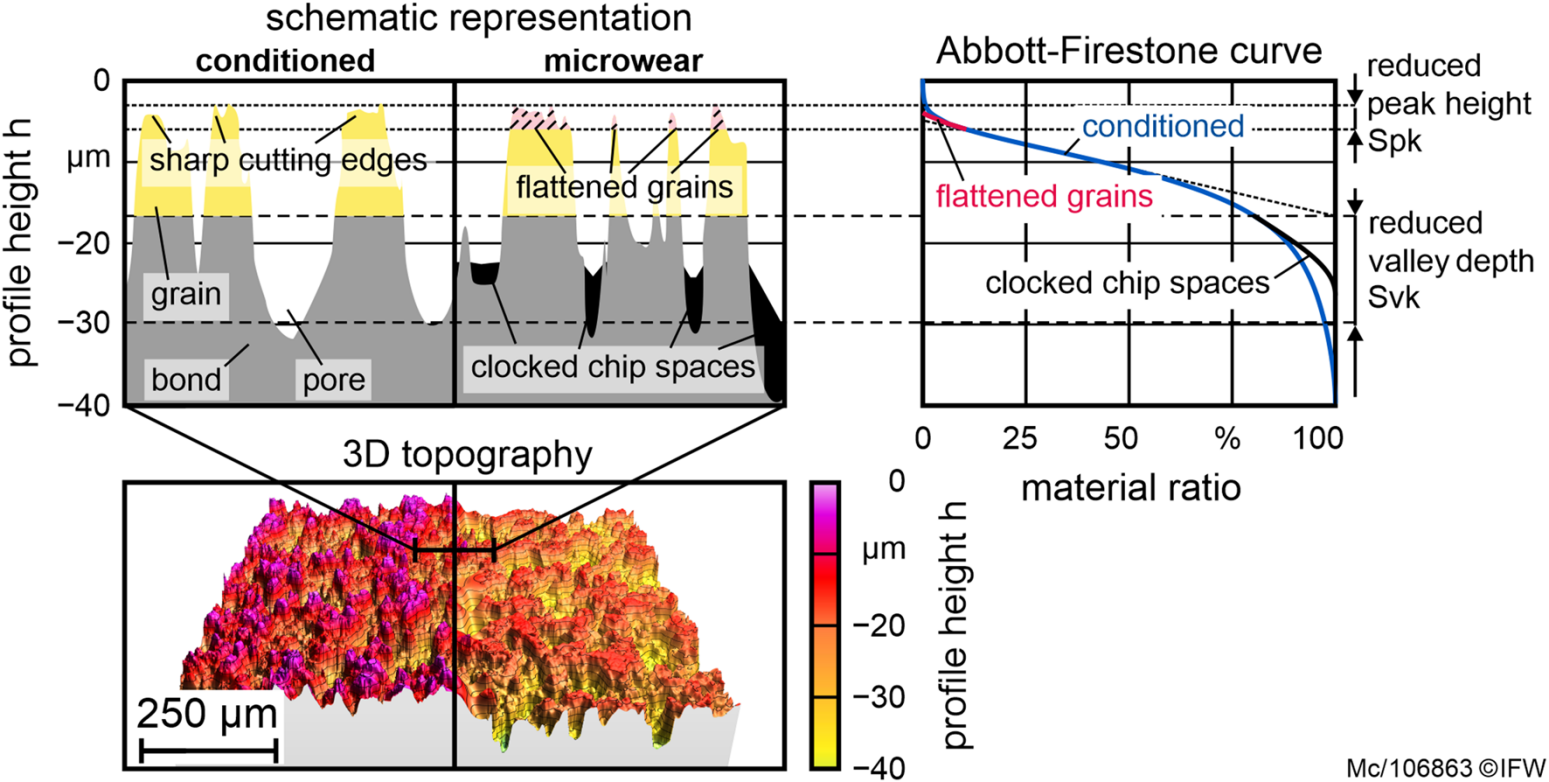
Methodology of microscopic wear evaluation by comparing the areal parameters Spk and Svk of the Abbott–Firestone curve of the worn and conditioned topography.

In the first test series, the influence of the conditioning process on the grinding layer topography is investigated without the influence of the grinding process. To generate the same worn initial state, the grinding layer is worn by grinding a thick film PcBN insert with *v*_*c*_ = 20 m/s, *v*_*fa*_ = 4 mm/min and *a*_*e*_ = 4 × 100 µm. According to prior investigations, these process parameters generate high profile wear. The cemented carbide support of the cutting insert causes welds on the grinding tool topography^8^. In this series of tests, the abrasive layer is conditioned by varying the grain size of the dresser *d*_*gd*_ and the circumferential speed of the dressing tool *v*_*rd*_. The conditioning infeed is kept constant at *a*_*ed*_ = 1 µm and 30 strokes are performed. A lower circumferential speed *v*_*c*_ = 15 m/s is chosen, which is adapted to the typical cutting speeds for less wear on the grinding tool without affecting the roughness of the ground workpiece^8,15^. At the different stages, 3D profiles of the worn topography of the abrasive layer are generated using a 3D white-light microscope InfiniteFocus G5 from Alicona (Fig. 3, bottom). The topography of the abrasive layer is described by the topography parameters of the Abbott-Firestone curve as depicted in Fig. 3. The areal functional parameters reduced peak height *Spk* and the reduced valley depth *Svk* are compared with the surface characteristics of the previous condition. A decrease in the reduced peak height with otherwise identical surface characteristics indicates a flattening of the diamond grain. A decrease in the reduced valley depth at the same core roughness depth is an indication of clogging of the grinding layer.

**Figure 4:**
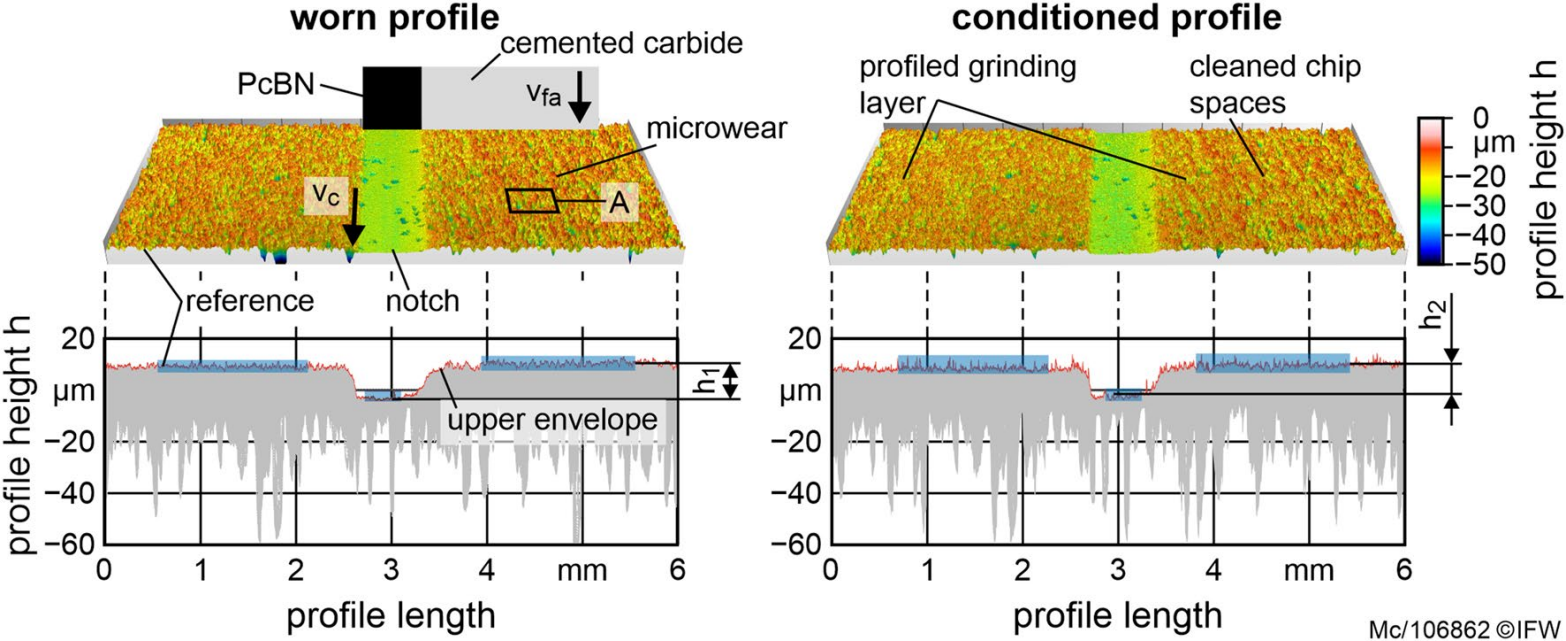
Methodology of measuring the topography and profile of the grinding layer.

Furthermore, the PcBN generates a circumferential notch with a depth of *h*_*1*_ = 10 µm (Fig. 4, top). The generated topography of the abrasive layer is described using the envelope of the 3D profile (Fig. 4, bottom) to evaluate the resulting change in the height of the notch *Δh* = *h*_*2*_—*h*_*1*_. Consequently, the specific conditioning material removal rate $Q_{sd}^{\prime }$ is calculable according to Eq. (1).

1 $$Q_{sd}^{\prime } = \frac{{\pi \cdot \Delta h \cdot \left( {d_{s} - b_{s} } \right)}}{{t_{d} }}$$

It describes the speed with which the grinding layer profile is restored. $Q_{sd}^{\prime }$ is calculated by determining the difference in notch depth *Δh* in the abrasive layer between worn profile *h*_1_ and conditioned profile *h*_2_. This height difference is multiplied by the ring area of the abrasive layer and divided by the width of cut of the conditioning tool *a*_*pd*_ and the total time of the conditioning process *t*_*d*_. In the present case, *a*_*pd*_ corresponds to the width of the abrasive layer *b*_*s*_. $Q_{sd}^{\prime }$ is varied under the same process parameters in test series 2. Hereby, the influence on the specific cutting power is investigated. The optimum value of $Q_{sd}^{\prime }$ lies in the minimum of the volume-specific cutting power $P_{c}^{\prime \prime \prime }$.

**Figure 5:**
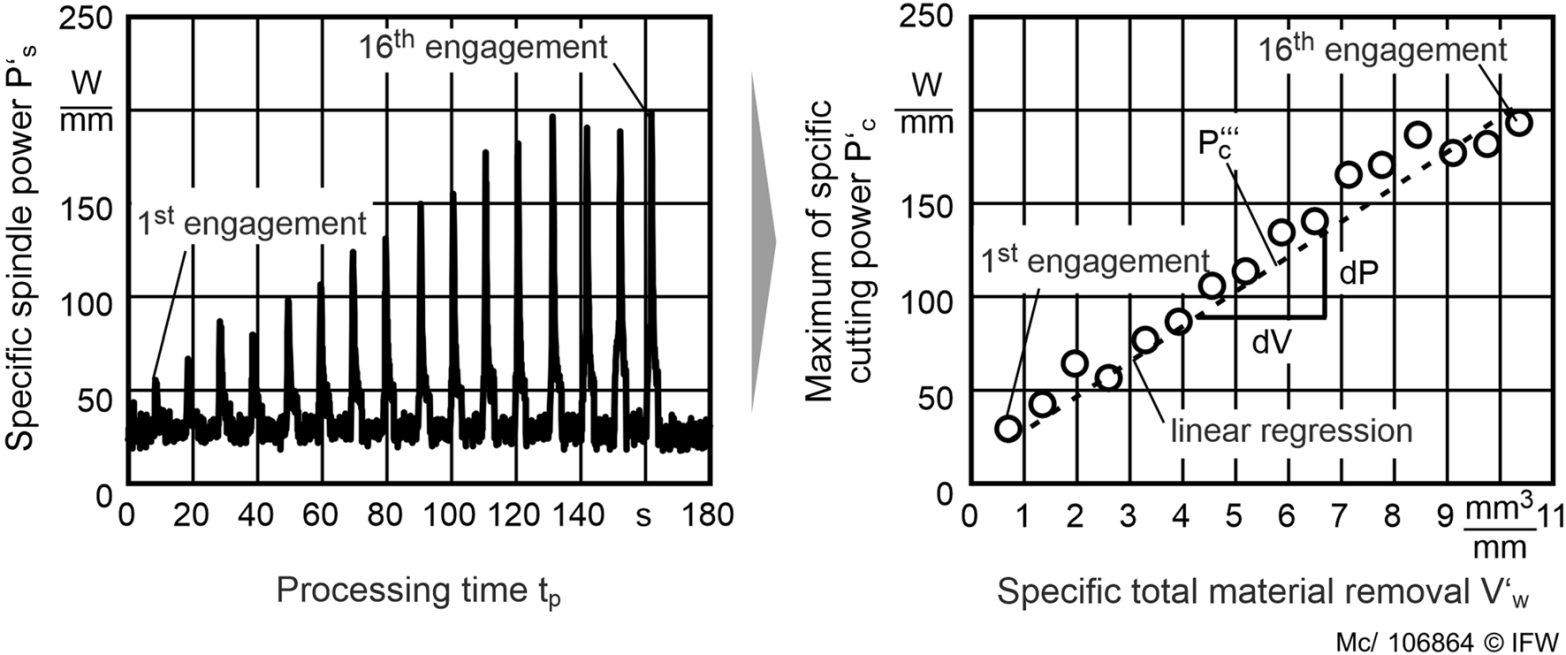
Methodology for determining the volume-specific cutting power.

In the second series of tests, the influence of the grinding layers’ topography and its interaction with the process parameters of the grinding process is determined. For these investigations, in-process conditioning is applied to the grinding process of PcBN inserts. A high feed rate *v*_*fa*_ = 12 mm/min and a low cutting speed corresponding to test series 1 is selected. According to the criteria of test series 1, this causes less wear on the grinding tool without affecting the roughness of the ground workpiece^8,15^. The parameter variation of the conditioning process is performed ceteris paribus the first test series (Table 1) and, in addition, the infeed of the conditioning tool per stroke *a*_*ed*_ is varied as shown in Table 2. In the process, the time course of the power consumption of the grinding spindle is determined using the Tyrolit Toolscope in process measurement technology according to^16^. This data recorder enables the recording of the spindle power and position of the axes of the machine tool. Therefore, the specific material removal $V_{w}^{\prime }$ is calculated from the position of the x-axis. $P_{s}^{\prime }$ is calculated by dividing *P*_*s*_ by the width of cut *a*_*p*_. The maximum cutting power per tool engagement $P_{c}^{\prime }$ is calculated by subtracting the idle power of the machine tool spindle from the maximum spindle power *P*_*s*_. The rise of the specific cutting power with increasing $V_{w}^{\prime }$, which is $P_{c}^{\prime \prime \prime }$, is an indicator for the microscopic wear rate of the grinding tool (Fig. 5).

**Table 1:** Factor levels of test series 2: determining the influence of the dressing parameters on the process parameters.

Test series	Parameter	Test point		
		− 1	0	+ 1
1, 2	*d*_*gd*_ (µm)	30	48	125
1, 2	*v*_*rd*_ (m/s)	1.5	2.2	3.0
2	*a*_*ed*_ (µm)	0.5	1.0	1.5

**Table 2:** Specification of ground PcBN workpieces.

Specification	Binder	Concentration of cBN (%)	cBN Particle size (µm)	Hardness (HV0.2)
A	TiN	55	2	2561 ± 92
B	TiN/TiC	75	2	3315 ± 78

$P_{c}^{\prime \prime \prime }$ is the slope of a linear regression of $P_{c}^{\prime }$ to $V_{w}^{\prime }$. As shown by^16^, the specific spindle power is highly correlated with the specific cutting energy, which depends not only on the chip formation but also on sliding in a grinding process. The flattening of the diamond grains, grain dulling, and weld build-up lead to an increased coefficient of friction between the grinding tool and workpiece. This increased coefficient of friction leads to an increase in the spindle power. Accordingly, a grinding process with the lowest possible volume-specific spindle power, e.g., a static level of $P_{c}^{\prime }$, is the objective of the investigations.

In each experiment, face-centered test plans with randomized test points are used. Both test series are performed with three different conditioning tools with varied grain sizes *d*_*gd*_ = 30 µm (#500 mesh), 48 µm (#320 mesh), and 125 µm (#120 mesh) of white corundum (Table 1). These are typical grain sizes that can be provided by the dressing tool manufacturer Saint-Gobain Abrasives GmbH. The circumferential speed of the conditioning tool is varied in three steps between *v*_*rd*_ = 1.5 m/s and 3 m/s. In the first series of tests, 30 strokes of the conditioning tool are performed consecutively. In the second series of tests, continuous dressing is performed. The dressing depths of cut per tool stroke are varied between *a*_*ed*_ = 0.5, 1.0, and 1.5 µm. The grinding tool is a cup grinding wheel with an outer diameter *d*_*s*_ = 400 mm. The abrasive layer has a width of *b*_*s*_ = 15 mm and consists of a vitrified diamond grain of grain size D15A, which corresponds to an average grain diameter *d*_*g*_ = 12 µm. An abrasive concentration C100 is used, which corresponds to a volume fraction of the diamond grain *C* = 25%.

In test series 2 two different specifications of thick film PcBN inserts with cemented carbide support are ground (Table 2): Specification A has a cBN content of 55%. The hardness of the material is determined at 2,561 ± 92 HV0.2. Specification B has a cBN content of 75% and a hardness of 3315 ± 78 HV0.2. The average particle size of cBN of both specifications is *d*_*cBN*_ = 2 µm. The workpieces are ground four times on each flank face with an infeed *a*_*e*_ = 50 µm. Therefore, 16 engagements to a total material removal $V_{w}^{\prime }$ = 10.4 mm^3^/mm are performed.

In all tests, a mineral oil is used as cooling lubricant with a flash point of 165 °C. The cooling lubricant is applied to the engagement zone between the workpiece and the grinding tool using needle nozzles with a flow rate of *Q *= 35 l/min.

## Results and discussion

### Conditioning of the grinding layer (test series 1)

**Figure 6:**
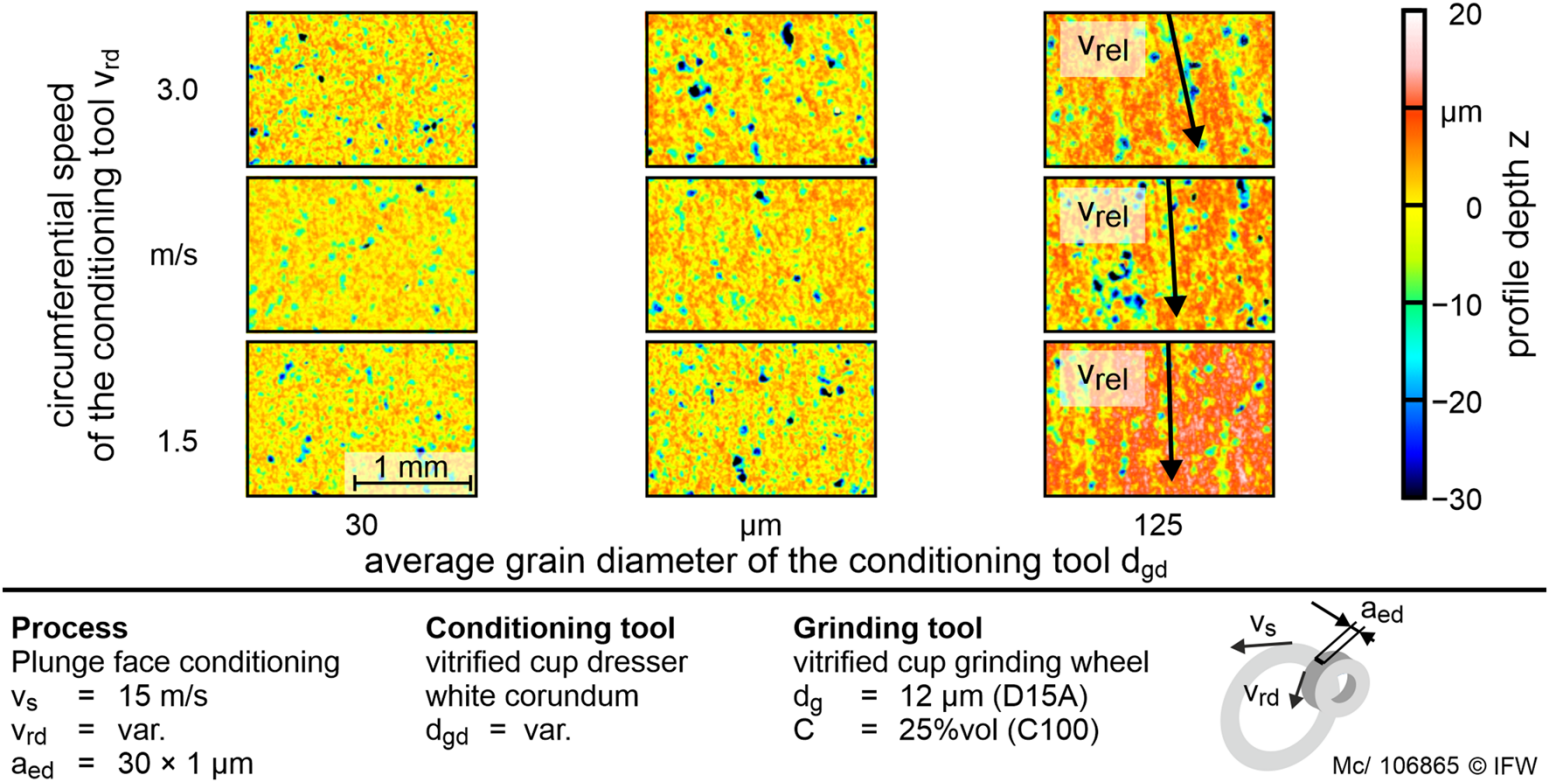
Influence of the circumferential speed and the average grain diameter of the cup conditioning tool on the grinding layer topography. The conditioning grain diameter d_gd_ = 125 µm generates deep groves on the topography.

To investigate the conditioning process of the grinding tool, the influence of the corundum grain size and the circumferential velocity of the conditioning tool on the generated topography of the grinding tool. The circumferential speed of the grinding tool is set to *v*_*sd*_ = 15 m/s. Figure 6 shows the resulting topographies for the input parameters mentioned.

With increasing grain size, an increase in grain protrusion of the diamond grains can be observed. The grain sizes *d*_*gd*_ = 30 µm and *d*_*gd*_ = 48 µm caused homogeneous material removal. Accordingly, the surfaces are characterized by randomly and homogeneously distributed peaks and valleys. However, the topographies at *d*_*gd*_ = 125 µm show deep grooves parallel to the direction of the relative speed *v*_*re*l_ between the conditioning tool and the grinding tool. These grooves are up to the depth of the diamond grain diameter. The elongated groove structure can be reduced by increasing the circumferential speed of the conditioning tool. Since the dressing tool is in contact with two contact zones with opposite directions of movement, the contact paths cross at the directional angle of *v*_*rel*_. At high rotational speeds of the conditioning tool, this leads to less overlap of the grooves on the grinding tool surface. However, a high inhomogeneity of material removal remains in the grinding layer using *d*_*gd*_ = 125 µm corundum grain size despite a high removal rate and high grain protrusion.

**Figure 7:**
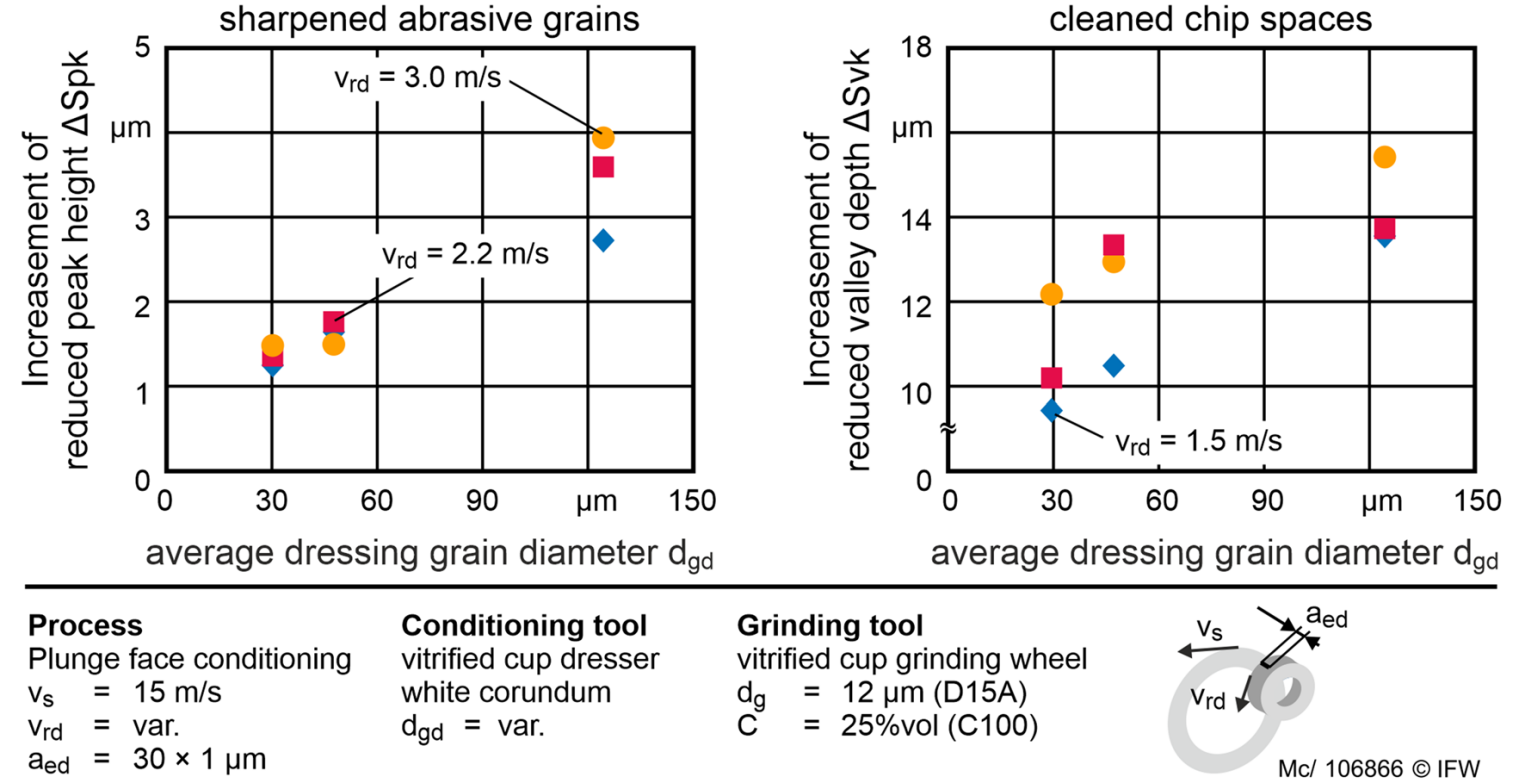
Influence of the average grain diameter of the conditioning tool on the surface parameters Spk and Svk. Its influence is five times greater in Svk than in Spk. With increasing grain diameter, both Spk and Svk increase.

These results are supported by the quantitative data of the reduced valley depth *Svk* and the reduced peak height *Spk*. Figure 7 depicts the influence of the grain size *d*_*gd*_ and the circumferential speed v_*rd*_ of the conditioning tool on the surface parameters of the grinding layer *Spk *and *Svk*. The influence of the circumferential speed *v*_*rd*_ is negligibly small for both surface parameters in the investigated range. In contrast, the influence of the grain diameter of the conditioning tool *d*_*gd*_ is significant but more than five times higher on the reduced valley depth *Svk* than on the reduced peak height *Spk*. The reduced peak height increases with increasing grain diameter of the white corundum. The corundum grains are flattening without further decreasing the bond. With a small corundum grain diameter, the influence on *Svk* is initially high. The grains of the conditioning tool can engage the bond material and set it back evenly. The diamond abrasive is harder than the corundum, while the bond has a lower hardness. If the size of the corundum grains is small, the diamond removes the corundum grains without the contact of the corundum grains on the bond. Therefore, the corundum is not able to remove the bond sufficiently. As the grain size increases, the corundum grains are less removed by diamonds and engage more with the bond from the abrasive layer. With a corundum grain diameter *d*_*gd*_ = 125 µm, single sharp diamond grains are released from the bond. The analysis of the grinding wheels topographies shows that diamond grains roll through the layer surface and appear as curl marks, respectively, the observed grooves on the surface of the abrasive layer. Although the large grain size leads to a high value of *Svk*, it also leads to an uneven dressing result. The increase in *Spk* is significant but small. This is because this parameter depends mainly on the tips of highly hard diamonds. The corundum does not significantly damage the diamond grains.

The evaluation of the influence of the conditioning parameters is built on a significance analysis regarding to a quadratic regression model. A quadratic regression model is chosen to consider a possible influence of both the kinetic energy *E*_*kin*_ = *m*·*v*^2^ and the cross-sectional area of the chip *A*_*cu*_ = f(*d*_*gd*_^*2*^) of the corundum grains. The results are shown in Table 3. Terms of significance *p* < 0.05 are stated significant and *p* < 0.01 are stated high significant. The analysis shows a significant influence of *d*_*gd*_ on the surface parameters *Spk* (*p*(*d*_*gd*_) < 0.01) and *Svk* (*p*(*d*_*gd*_) < 0.05). For $Q_{sd}^{\prime }$ the influences of *d*_*gd*_ (*p* < 0.01), *d*_*gd*_^2^ (*p* < 0.01) and *v*_*rd*_ (*p* < 0.05) are significant. Hence, $Q_{sd}^{\prime }$ is influenced by the diameter of the corundum grain, the cross-sectional area of the chip, and the circumferential speed of the conditioning tool. The terms of significance only allow for the identification of a generalized influence of the conditioning parameters on the surface of the grinding layer.

**Table 3:** Term significance of the conditioning parameters on Spk, Svk and $Q_{sd}^{\prime }$. Lower values are more significant.

Term	Term significance *p*					
	Const	*d*_*gd*_	*v*_*rd*_	*d*_*gd*_·*v*_*rd*_	*d*_*gd*_^2^	*v*_*rd*_^2^
*Spk*	**0.035**	**0.006**	0.495	0.862	0.445	0.561
*Svk*	**0.00**	**0.034**	0.651	0.914	0.421	0.623
$Q_{sd}^{\prime }$	**0.014**	**0.001**	**0.043**	0.551	**0.002**	0.599

Significant values are in [bold].

The influence of *d*_*gd*_ and *v*_*rd*_ and the specific conditioning material removal rate $Q_{sd}^{\prime }$ can be described using a regression model according to Eq. (2).

2 $$Q_{sd}^{\prime } = \left( {5.4\frac{{d_{dg} }}{{\upmu {\text{m}}}} - 0.03\frac{{d_{gd}^{2} }}{{\upmu {\text{m}}^{2} }} - 33.4\frac{{v_{rd} \cdot {\text{s}}}}{{\text{m}}}} \right) \cdot 10^{ - 3} \frac{{{\text{mm}}^{2} }}{{{\text{mm}}\;{\text{s}}}}$$

For low and high grain diameters, the $Q_{sd}^{\prime }$ in the investigated grain size range is reduced by up to 75% compared to the maximum of $Q_{sd}^{\prime }$. The influence *v*_*rd*_ is more than six times higher than the influence of the grain size. In summary, the conditioning process of the examined grinding tool achieves a local optimum $Q_{sd}^{\prime }$ = 0.14 mm^3^/mm s for profiling at *d*_*gd*_ = 48 µm and *v*_*rd*_ = 1.5 m/s. Higher values can be reached by increasing the feed frequency *f*_*d*_, since more strokes are performed per second. The regression model is used to calculate $Q_{sd}^{\prime }$ in test series 2. If the corundum grain diameter is reduced, the surface characteristics *Spk* and *Svk* can be reduced. For this reason, the effects of these correlations on the grinding process are to be investigated in test series 2.

### Influence of in-process conditioning on the grinding process (test series 2)

**Figure 8:**
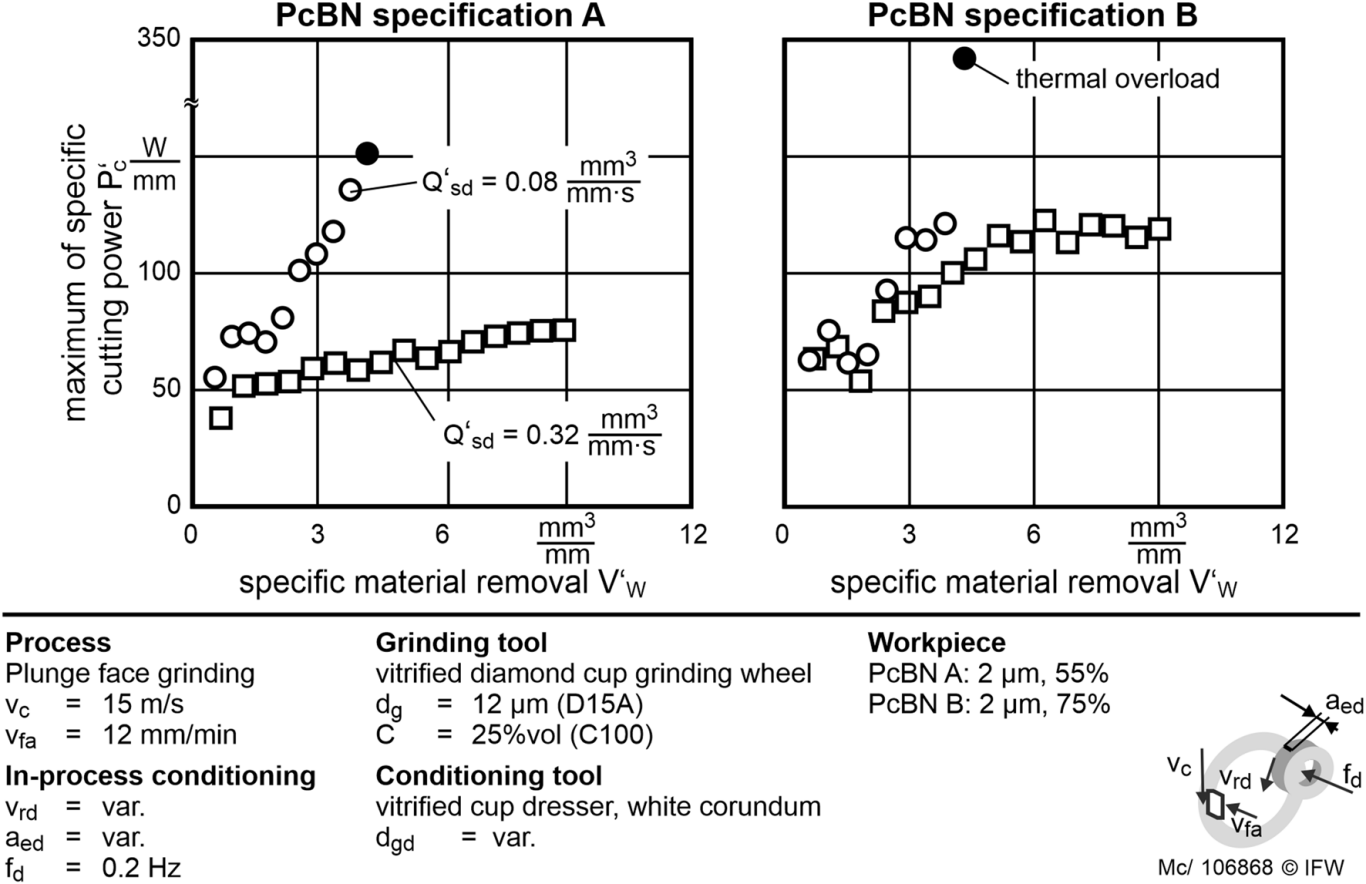
Influence of ${\text{Q}}_{{{\text{sd}}}}^{\prime }$ on the maximum specific spindle power with 16 grinding tool engagements. At low ${\text{Q}}_{{{\text{sd}}}}^{\prime }$ the tests had to be stopped because of thermal overload.

In this test series, the influence of the conditioning parameters on tool wear in in-process conditioning is investigated. The grinding is carried out with constant grinding parameters and varied specific conditioning material removal rates $Q_{sd}^{\prime }$. Generally, the spindle power continuously increases, and experiments are carried out until the desired cutting volume is reached. Figure 8 shows examples for both PcBN specifications and the grinding process. All experiments are on an equal level regarding the specific cutting power $P_{c}^{\prime }$ during the first engagement. At this point, the grinding wheel topography is in the same unworn initial state. The specific cutting power increases slightly for PcBN specification A with low cBN content in conditioning with $Q_{sd}^{\prime }$ = 0.32 mm^3^/mm s. For the same value of $Q_{sd}^{\prime }$ at PcBN specification B with medium cBN content, a higher increase of $P_{c}^{\prime }$ can be observed. Specification B is harder than specification A. Higher hardness leads to higher resistance to mechanical wear. Hence, micro-wear is higher in the grinding of PcBN B than in the grinding of PcBN A.

During in-process conditioning with $Q_{sd}^{\prime }$ = 0.08 mm^3^/mm s, a spontaneous thermal overload in the contact area is detected by smoke. This indicates that the flash point of the lubricant is reached in the contact zone. The respective test is canceled to prevent the burning of the coolant. The grinding process with a thermal overload, i.e. high $P_{c}^{\prime \prime \prime }$, has to be stopped consistently prematurely. This overload occurs more frequently at a small grain diameter *d*_*gd*_ = 30 µm. For these experiments, the regression model results in low values of $Q_{sd}^{\prime }$ = 0.08 mm^3^/mm s. Also, low values of *Svk* are investigated in test series 1 of the experiment. Because of this limitations, adequate conditioning in the process may not be guaranteed with the conditioning tool using *d*_*gd*_ = 30 µm. Therefore, the sharpening rate of the abrasive layer is too low in in-process conditioning. Both measured values *Spk* and *Svk* are small in test series 1 for *d*_*gd*_ = 30 µm compared to wider grain diameters. It can be stated that a low grain protrusion and a small grain size lead to increased leveling of the abrasive layer surface: at small grain sizes, the surface is too evenly conditioned, and insufficient sharp cutting edges remain on the surface. Furthermore, with a large corundum grain diameter (*d*_*gd*_ = 125 µm) an uneven profiling of the abrasive layer was observed even during in-process conditioning, so that a plane flank face of the PcBN insert cannot be ensured (Figs. 6, 7).

**Figure 9:**
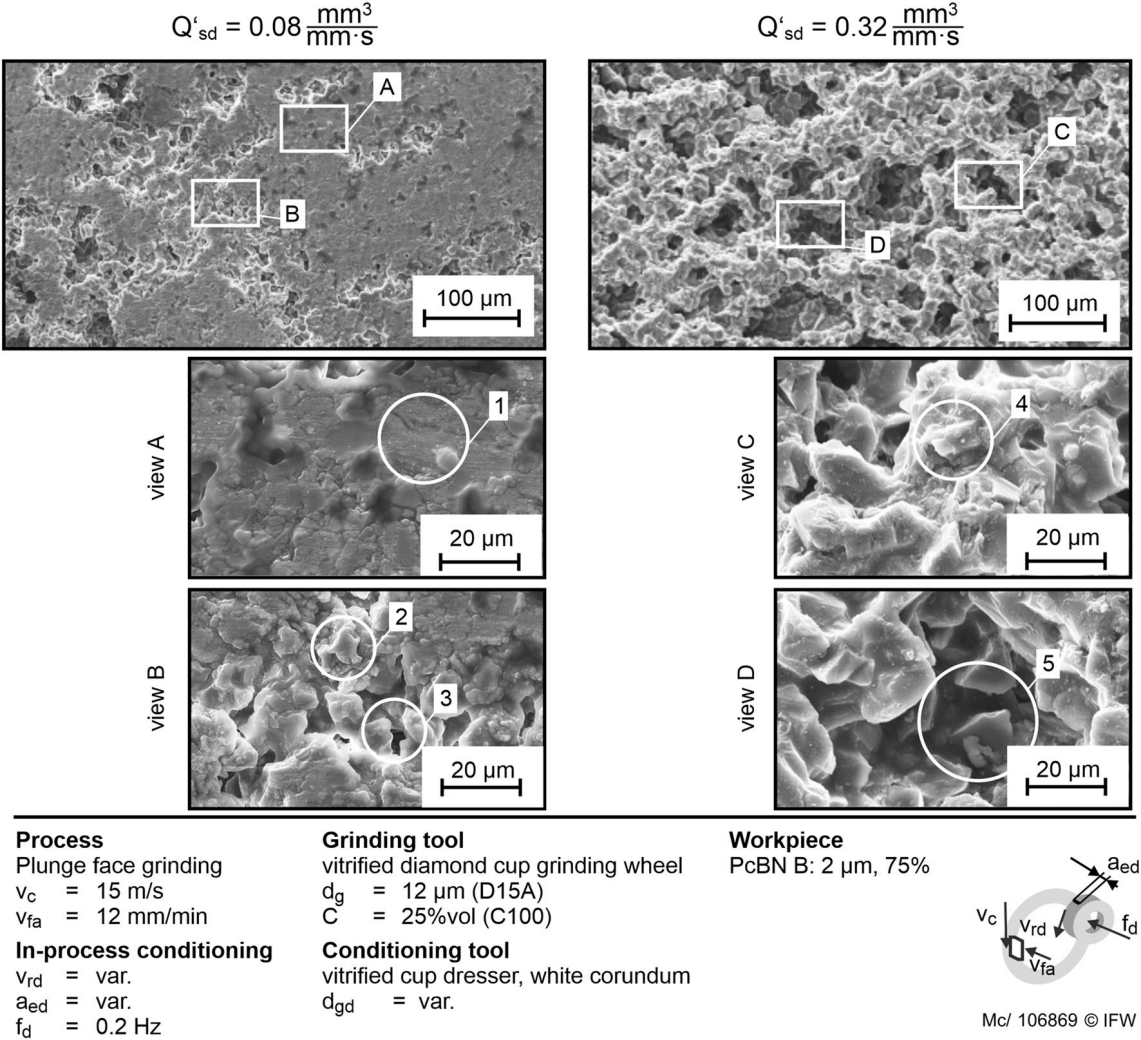
SEM images of the abrasive layer after grinding with in-process conditioning at ${\text{Q}}_{{{\text{sd}}}}^{\prime }$ = 0.08 and 0.32 µm^3^/mm s.

To evaluate the topography of the surface layer and verify the presented results, SEM images of both test runs $Q_{sd}^{\prime }$ = 0.08 mm^3^/mm s and $Q_{sd}^{\prime }$ = 0.32 mm^3^/mm s are taken (Fig. 9). The topography after grinding the same PcBN specification with in process conditioning at $Q_{sd}^{\prime }$ = 0.08 mm^3^/mm s is shown left. The topography is clogged in large areas. Detail 1 shows a bound embedded abrasive grain with nearly no grain protrusion. The diamond grains are dulled in areas with low amount of clogging, as shown in detail 2 and 3. The low value of $Q_{sd}^{\prime }$ = 0.08 mm^3^/mm s leads to an insufficient removal of dulled abrasive grains. Due to the insufficient sharpening of the abrasive layer, the grain protrusion drops until the grains are totally embedded into bond particles. Therefore, a smaller amount of coolant is provided to the contact zone. Grinding debris remain in the surface rather than being removed by the coolant. Consequently, the amount of rubbing increases until a thermal overload occurs. The mechanisms to describe the intensity of the dressing discussed in^12^ can also be observed in plunge face grinding. A high conditioning intensity, described by $Q_{sd}^{\prime }$, leads to more cutting edges. The conditioning process removes dulled abrasive grains and restores the topography of the abrasive layer to its initial state. Therefore, the influence of microscopic wear is lowered by high values of $Q_{sd}^{\prime }$.

The topography is characterized by many abrasive grains with sharp cutting edges (detail 4), a high grain protrusion, and deep pores (detail 5). Because of the sharp cutting edges, the abrasive grains can cut PcBN effectively. The high grain protrusion and deep pores result in sufficient wetting of the surface with coolant and a low amount of rubbing between the bond and the workpiece surface. Therefore, the grinding power increases up to the 16th engagement only to a small extent.

**Figure 10:**
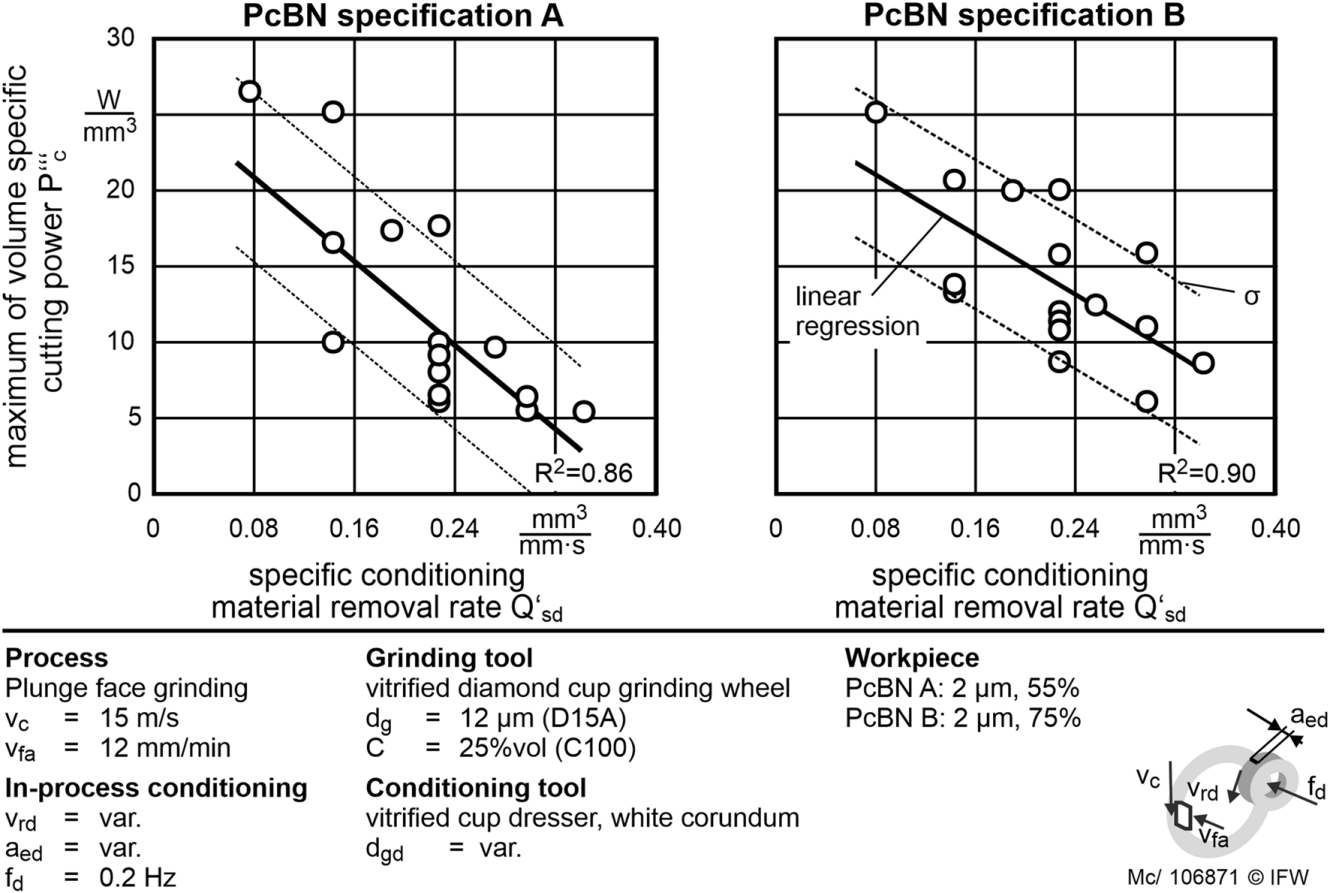
Linear regression model and experimental data of ${\text{P}}_{{\text{c}}}^{\prime \prime \prime }$ depending on ${\text{Q}}_{{{\text{sd}}}}^{\prime }$ for both PcBN specifications. ${\text{P}}_{{\text{c}}}^{\prime \prime \prime }$ is reduced by increasing ${\text{Q}}_{{{\text{sd}}}}^{\prime }$.

Subsequently, the respective conditioning material removal rates are calculated for both PcBN specifications using the relation shown in Eq. (2). These are compared to the determined volume-specific spindle power $P_{c}^{\prime \prime \prime }$. There is a highly correlating linear relationship between $Q_{sd}^{\prime }$ and $P_{c}^{\prime \prime \prime }$ (Fig. 10). According to this model, it is possible to adjust the removal rate with in-process conditioning the reduced the micro-wear even when grinding PcBN. Therefore, microscopic tool wear is reduced, resulting in a porous grinding layer topography with high grain protrusion and sharp cutting edges. The sharp cutting edges increase the material removal per grain until the abrasive grains are broken out of the abrasive layer. Consequently, the macroscopic tool wear is reduced. Therefore, the effort in profiling the grinding tool is reduced and a smaller number of abrasive grains must be removed by the conditioning process.

## Conclusions

These investigations aimed to reduce the microscopic tool wear and the specific grinding power in plunge face grinding of PcBN. The purpose is a regression model-based parameterization of in-process conditioning with white corundum cup wheels in plunge face conditioning. For the optimization of the conditioning process, nonlinear regression models were used, whose input variables are based on face-centered test plans. In the first test series, both regression models for the influence of the conditioning parameters on the grinding tool surface and qualitative descriptions of the conditioning are derived. The results of the first test series are input for the second test series. Here, in-process conditioning is performed by grinding two PcBN specifications.

An indicator of microscopic wear of the grinding tool is $P_{c}^{\prime \prime \prime }$. Lower values of $P_{c}^{\prime \prime \prime }$ represent lower wear rates of the grinding layer. By increasing the specific conditioning material removal rate $Q_{sd}^{\prime }$, $P_{c}^{\prime \prime \prime }$ can be reduced almost to zero. The micro-wear of the vitrified bonded diamond grinding tool decreases significantly with increasing $Q_{sd}^{\prime }$. Grinding of PcBN is possible by in-process conditioning with $Q_{sd}^{\prime }$ = 0.32 mm^3^/mm s without significant micro-wear on the grinding tool. This eliminates the need for time-consuming conditioning in the non-productive time and reduces the total energy consumption of the process.

The grain size of the corundum conditioning tool *d*_gd_ has a significant nonlinear influence on the reduced peak height *Spk* and the reduced valley depth *Svk* of the topography of the grinding layer. Here, the influence of *d*_gd_ is more than five times higher on* Svk *than on *Spk*. In the investigated corundum grain size interval of *d*_gd_= 30 – 125 µm, Spk increases with *d*_gd_ Thus, an increase in *d*_gd_ leads to an increase in grain protrusion. More diamond grains can cut PcBN and therefore the grinding process is more effective. The use of larger grain sizes is not recommended for the diamond grain size examined of *d*_g_= 12 µm, as grooves and uneven structures affect the surface of the abrasive layer.

The specific conditioning removal rate $Q_{sd}^{\prime }$ reaches a maximum at *d*_gd_= 48 µm. The circumferential speed of the conditioning tool *v*_rd_ was identified as the second significant influencing factor on the dressing material removal rate. A reduction in *v*_rd_ leads to an increase in $Q_{sd}^{\prime }$ without significantly influencing the microscopic topography of the grinding layer.

The results of this investigation have shown that in-process conditioning should be performed with medium grain sizes from #220 to #320 US-mesh with low conditioning tool circumferential speed when grinding PcBN inserts. The selection of these parameters allows sufficient conditioning of the grinding layer to enable the grinding of PcBN with low microscopic and macroscopic wear on the grinding tool. This significantly reduces the specific cutting energy in the grinding of PcBN. Thus, an in-process conditioning process with a high conditioning material removal rate enables a considerable potential for resource savings in grinding PcBN.

## Data Availability

On request.
